# Video Capsule Endoscopy: A Tool for the Assessment of Small Bowel Transit Time

**DOI:** 10.3389/fmed.2016.00006

**Published:** 2016-02-10

**Authors:** Reza A. Hejazi, Mohammad Bashashati, Mohammed Saadi, Zuber D. Mulla, Irene Sarosiek, Richard W. McCallum, Marc J. Zuckerman

**Affiliations:** ^1^Division of Gastroenterology, Department of Medicine, Mayo Clinic, Jacksonville, FL, USA; ^2^Division of Gastroenterology, Department of Medicine, Texas Tech University Health Sciences Center, El Paso, TX, USA; ^3^Division of Gastroenterology, Department of Medicine, Temple University, Philadelphia, PA, USA; ^4^Department of Obstetrics and Gynecology, Texas Tech University Health Sciences Center, El Paso, TX, USA; ^5^Department of Public Health, Texas Tech University Health Sciences Center, Lubbock, TX, USA

**Keywords:** video capsule endoscopy, small intestine transit time, wireless motility capsule

## Abstract

**Purpose:**

Video capsule endoscopy (VCE) is a procedure that uses a wireless camera to take pictures of the gastrointestinal (GI) tract. A wireless motility capsule (WMC) of a similar size has been developed, which measures pH, pressure, and temperature and can be used to assess regional and total GI transit times. VCE could also potentially be used as a tool for measuring small bowel transit time (SBTT).

**Methods:**

This study was designed to obtain SBTT from VCE and compare it with historical data generated by WMC. Gastric transit time (GTT) was also measured. Patients were included if the indication for VCE was either iron deficiency anemia (IDA) or overt obscure GI bleed (OOGIB), and they did not have any known motility disorder. Results from VCE were also compared in diabetic vs. non-diabetic patients.

**Results:**

There were a total of 147 VCE studies performed, including 42 for OOGIB and 105 for IDA. Median GTT and SBTT were 0.3 and 3.6 h, respectively. The overall median GTT and SBTT were 0.3 and 3.6 h, respectively, in the IDA group compared with 0.3 and 3.4 h in the OOGIB group. When compared with WMC, the GTT and SBTT were significantly faster in both groups (GTT: 3.6 h and SBTT: 4.6 h). The median GTT and SBTT were not significantly different in diabetics vs. non-diabetics [GTT: 17.5 vs. 18.0 min (*P* = 0.86) and SBTT: 3.9 h (237 min) vs. 3.8 h (230 min), respectively (*P* = 0.90)].

**Conclusion:**

SBTT as measured using VCE is not significantly different in OOGIB compared with IDA. Both GTT and SBTT are significantly faster as assessed by VCE, which is initiated in the fasting state, compared with WMC measurement, which is initiated after a standard meal. In summary, VCE could potentially be used for measuring SBTT in the fasting state.

## Introduction

Studying small bowel transit time (SBTT) is complex because of the anatomical organization of this organ. While the transit time and motility patterns of the small intestine are less appreciated compared with other segments of the gastrointestinal (GI) tract in our clinics, assessment of these parameters might be helpful in patients with pseudo-obstruction, abdominal pain of unknown cause, a number of metabolic disorders, and possibly small intestinal bacterial overgrowth (SIBO) (1, 2).

Available tools for the assessment of small intestinal motility are suffering from several limitations making them inaccurate for both clinical and research purposes. Among them, the oldest method, i.e. radiographic barium study or small bowel follow-through, provides structural information without an accurate transit measurement. Small intestinal manometry can only study the upper part of the small intestine. Moreover, with this technique, the insertion of the catheter may interfere with the physiologic motility patterns. Radiopaque markers are mainly suitable for the study of colonic and whole gut transit. Hydrogen breath test measures orocecal transit time rather than the pure small intestinal transit time, and its results may be misinterpreted in the presence of SIBO (1, 3).

Scintigraphic methods, involving isotope-labeled liquids or meals, expose the patients to radiation and their accuracy in the measurement of SBTT is affected by the gastric emptying component.

Wireless motility capsule (WMC), which uses changes in pH as the landmarks, is currently the method of choice for studying the SBTT. The capsule is ingested immediately after consuming a calorie meal in order to induce a “fed state” setting. In 5–10% of cases, the determination of SBTT is not possible with this technique due to difficulties in interpretation of entrance into the cecum (4). Moreover, WMC has demonstrated different transit values based on age, gender, and country where the study is performed and testing protocol (5).

On the other hand, video capsule endoscopy (VCE), which provides visualization of the GI tract through wireless transmission of images from a disposable capsule to a data recorder worn by the patient, enables us to observe the luminal surface of the small intestine and to also study small transit time. As opposed to the standard endoscopy procedures, the images do not come from a camera being driven into the digestive tract, but rather images that are captured as the capsule is carried by peristalsis through the gut. The first capsule model for the small intestine was approved by the Food and Drug Administration (FDA) in 2001. VCE is mainly a modality to diagnose small intestinal pathology (6–10). This essentially gives a direct examination of the entire length of the small bowel in a non-invasive manner (6, 8, 11). Moreover, small intestinal water content and intraluminal secretion as the lubricators of the tract can be studied with this method making it a suitable tool in both clinical and research settings (12).

Here, we hypothesize that although VCE was essentially designed to find organic abnormalities in the small intestine, the time elapsed from its entrance to the duodenum to its appearance in the cecum may be measured as small intestinal transit time. To test our hypothesis, SBTT was analyzed in a group of patients without GI motility symptoms in whom VCE were performed and then compared with data from historical healthy control subjects who have undergone WMC studies. In addition, we measured gastric transit time (GTT), which can be a confounding variable for assessing SBTT with the VCE method.

## Materials and Methods

Our study protocol was approved by the Institutional Review Board for the Protection of Human Subjects at Texas Tech University Health Sciences Center at El Paso, TX, USA.

### Measurement of the GI Transit Times

This study included 147 patients with obscure overt GI bleeding (OOGIB) or unexplained iron deficiency anemia (IDA) with capsule endoscopy (PillCam, Given Imaging Ltd., Israel). These patients had no history of any GI motility disorder. GTT and SBTT were measured. Results from VCE were then compared with WMC (SmartPill) published data from 66 healthy controls (13).

All patients had at least one negative upper and lower GI endoscopy before being referred for VCE examination. Exclusion criteria were confirmed or suspicion of GI motility disorders and the use of medications, which affect GI transit.

Before the VCE study, each patient was fasted overnight. Two hours after swallowing the video-capsule, patients were allowed to drink clear liquids and after 4 h, they were allowed to eat a light lunch. All VCE were reviewed by an expert (MZ) using the PillCam SB^®^ capsule endoscopy system (Given Imaging Ltd.). Images were viewed using the Rapid Reader (version 3.1) using either a single view or dual view mode, which had a maximal speed of 40 frames/s. Each VCE was analyzed for GTT and SBTT. The GTT was defined as the time of the first gastric image to the first duodenal image. SBTT was defined as the time from the first duodenal image to the first cecal image.

As mentioned above, GTT and SBTT values for patients with OOGIB, IDA, and the overall group (patients who had either OOGIB or IDA) were compared with the published transit values for the WMC method from 66 healthy controls that were reported by Sarosiek et al. (13). In that study, WMC was swallowed after ingestion of a standardized meal of 120 g Eggbeaters radiolabeled with technetium 99m sulfur colloid, two pieces of bread with jam, and an additional 120-cc of water (total calorie: 255 kcal). Subjects completed the meal within 10 min, and the capsule was ingested immediately after with up to 50 cc water. Patients had previously been fasting overnight. Pressure, pH, and temperature were continuously measured by the capsule and recorded by a portable receiver worn on the waist or suspended on a lanyard placed around the neck. None of the subjects had dyspeptic symptoms or known gastroparesis, and none of them had suspected obstruction or any other reason for prolonged capsule transit times (13).

### Statistical Analysis

Data were analyzed using SAS 9.3 software (SAS Institute, Inc., Cary, NC, USA) and OpenEpi (Open Source Epidemiologic Statistics for Public Health) Version 3.03a (www.openepi.com). Patients whose indication for capsule endoscopy was OOGIB were compared with those whose indication was IDA. Differences in the distributions of age, race/ethnicity, gender, and SBTT by indication were compared using a two-sample *t*-test, chi-square test, or Fisher’s exact test as appropriate. Results were considered statistically significant if the *P*-value was 0.05 or less.

A Pearson correlation coefficient was calculated to determine the strength of the association between GTT and SBTT. The null hypothesis was that rho, the correlation coefficient in the population, was 0. The null hypothesis was rejected if the *P*-value was 0.05 or less. Two multiple linear regression models were fit. In the first model, the outcome variable was GTT, while SBTT was the outcome in the second model. Patients who had a missing value for GTT were excluded from the regression analysis of SBTT. Four independent variables were entered in the regression models: indication (OOGIB vs. IDA), age (a continuous variable), gender, and race/ethnicity (entered using two dummy variables). Tolerances were calculated to determine if collinearity was present.

Sarosiek et al. (13) reported median rather than mean transit values. They also reported the 25th and 75th percentiles, thereby allowing the calculation of the interquartile range (75th percentile − 25th percentile). We assumed that the GTT and SBTT of the 66 historical controls followed bell-shaped distributions. This assumption allowed us to treat the medians that were reported by Sarosiek et al. as means and calculate the SD of the transit statistic using the well-known relationship, SD = interquartile range/1.35. Once the SDs were calculated from the results published by Sarosiek et al., two-sample *t*-tests were then performed comparing our mean GTT and SBTT values with the published historical data. A significance level of 0.05 was specified.

Within our patient population, median GTT and SBTT values in diabetics were compared to their respective values in the non-diabetics using Wilcoxon two-sample tests with a significance level of 0.05.

## Results

### Transit Time Measured by VCE

There were a total of 147 VCE studies performed, including 42 for OOGIB and 105 for IDA. Mean age (±SD) in the VCE group was 57.9 ± 14.2 years. Ninety-four (64%) patients were female and 135 (92%) were Hispanic. Demographic characteristics of the studied patients are shown in Table 1. Based on VCE, no patient had active bleeding.

**Table 1:** **Characteristics of 147 patients with overt obscure gastrointestinal (GI) bleeding (OOGIB) or iron deficiency anemia/occult obscure GI bleeding (IDA) who underwent video capsule endoscopy**.

Characteristic	OOGIB (*n* **=** 42)	IDA (*n* **=** 105)	All (*n* **=** 147)	*P* (OOGIB vs. IDA)
Age (years)^a^	57.0 (13.8)	58.2 (14.4)	57.9 (14.2)	0.66
Race/ethnicity^b^				1.0
Hispanic	39 (92.9)	96 (91.4)	135 (91.8)	
Non-Hispanic White	3 (7.1)	7 (6.7)	10 (6.8)	
Black	0 (0)	2 (1.9)	2 (1.4)	
Gender^b^				<0.0001
Female	15 (35.7)	79 (75.2)	94 (64.0)	
Male	27 (64.3)	26 (24.8)	53 (36.1)	
SBTT (h)^a^	3.7 (1.7)	3.8 (1.6)	3.8 (1.6)	0.70
	*n* = 34	*n* = 87	*n* = 121	

*^a^Mean (SD)*.

*^b^Number (percent)*.

**Figure 1:**
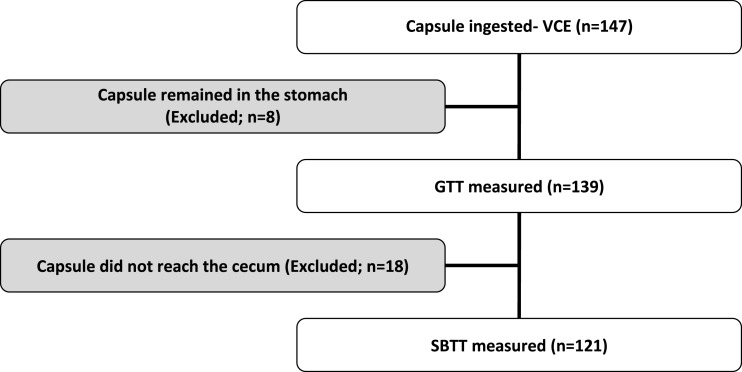
**Flow diagram of patients included for gastric transit time (GTT) and small bowel transit time (SBTT) analysis**.

The video endoscopy capsule did not reach the duodenum by the end of the study in 8 of 147 patients (5.4%). The capsule reached the cecum in 121/147 (82.3%) of patients. Therefore, GTT and SBTT were analyzed in 139 and 121 patients, respectively (Figure 1).

Selected patient characteristics by indication are presented in Table 1. Approximately 36% of the OOGIB group vs. 75.2% of the IDA group were females (*P* < 0.0001). In multiple linear regression analyses, no associations were detected between the outcomes of GTT and SBTT and the following four predictors: indication for VCE (OOGIB vs. IDA), age, race/ethnicity, and gender. There was a weak inverse linear association between GTT and SBTT among the 121 patients for whom both values were available (*r* = −0.19, *P* = 0.04).

Of those with available information regarding their diabetic status (i.e., 40 diabetics vs. 87 non-diabetics), the median GTT values were 17.5 min and 18.0 min, respectively (Wilcoxon two-sample test *P* = 0.86). For SBTT, 37 diabetics were compared with 83 non-diabetics. The median SBTT values in diabetics and non-diabetics were 237.0 and 230.0 min, respectively (Wilcoxon two-sample test *P* = 0.90).

### The Comparison between VCE and WMC

Median GTT measured by WMC was 3.6 h, while median SBTT was 4.6 h when measured by WMC (Table 2). Median GTT measured by VCE (in all patients combined) was significantly faster than when measured by WMC (0.3 vs. 3.6 h, *P* < 0.0001). Median SBTT as measured by VCE (in all patients combined) was also significantly faster compared with WMC (3.6 vs. 4.6 h, *P* = 0.0005) (Table 2).

**Table 2:** **Gastric (GTT) and small bowel (SBTT) transit times measured by video capsule endoscopy (VCE) compared to wireless motility capsule (WMC) historical values retrieved from a study by Sarosiek et al**.

Transit time (h)		VCE			WMC (HC)
		OOGIB	IDA	All	
GTT	Number of patients	41	98	139	66
	Median (25th percentile, 75th percentile)	0.3 (0.2, 0.8)^†^	0.3 (0.2, 0.5)^†^	0.3 (0.2, 0.6)^†^	3.6 (3.0, 4.2)
SBTT	Number of patients	34	87	121	66
	Median (25th percentile, 75th percentile)	3.4 (2.4, 4.9)^††††^	3.6 (2.6, 4.9)^†††^	3.6 (2.5, 4.9)^††^	4.6 (4.0, 5.9)

*HC, healthy control; IDA, iron deficiency anemia; OOGIB, obscure-overt gastrointestinal bleeding*.

*^†^*P* < 0.0001 for the comparison of OOGIB with HC, IDA with HC, and All with HC*.

*^††^*P* = 0.0005 for the comparison of All with HC*.

*^†††^*P* = 0.001 for the comparison of IDA with HC*.

*^††††^*P* = 0.003 for the comparison of OOGIB with HC*.

*P-values are from two-sample *t*-tests comparing means rather than medians (see Statistical Analysis for details)*.

## Discussion

The assessment of GI transit time with VCE has always been of interest and is noted on all procedure reports. In this study, we have shown that capsule endoscopy, which is mainly indicated for the visualization of small intestinal pathology, may alternatively be used for the measurement of SBTT. GTT and SBTT using VCE were not significantly different in OOGIB patients compared with IDA. However, both GTT and SBTT were significantly faster with VCE compared with WMC measurements.

Since there were no difference in the GI transit times between patients with OOGIB and those with IDA, and they had no known GI motility disorder, these patients could be considered as similar to healthy control population from a motility perspective. The difference between transit times measured by VCE and WMC is most likely explained by the different patients’ statuses, namely fasting vs. fed. VCE is performed in the fasting state when phase III migratory motor complexes regularly sweep the GI tract, while WMC is performed after a standardized solid meal where initiation of the migratory motor complex is delayed until gastric emptying of solids is achieved (14).

In a previous study from Spain on VCE that measured transit times in 89 patients, the mean GTT was approximately 23 min, while the mean SBTT was 283 min. No significant associations were found between gastric and intestinal transit times with the age and sex of studied subjects (15). VCE-recorded GTT (18 min) and SBTT (216 min) in our study are in the ranges reported in the above-mentioned study.

In another study which was designed to investigate the effect of different bowel preparations on GTT and SBTT measured by VCE, the median GTT was 25, 34.7, and 35 min among patients who were prepared with liquid diet, sodium phosphate, and polyethylene glycol, respectively. Mean SBTT among patients prepared with liquid diet, sodium phosphate, and polyethylene glycol was 264.4, 296.7, and 291.3 min, respectively. Again, GTT and SBTT values in this study were similar to our findings (16). In the same study, the capsule reached the cecum in approximately 83.6% of the studied patients. This is also in agreement with our study, as in 82.3% of our patients, the capsule reached the cecum and we could successfully measure SBTT (16).

Another retrospective study analyzed the association between the diagnostic yield of small bowel VCE and the SBTT. Based on this study, the cecum was reached in 82% of all procedures (17). Although all patients had received prokinetics before the procedure, the overall median small SBTT was 246 min (17), which was again in agreement with the findings of our study.

Current examinations done with extended battery-life capsules of 12 h have higher success rates in reaching the cecum.

The transit times measured by VCE in diabetic vs. non-diabetic patients have not been consistent in different studies. While we showed no difference between SBTT and GTT values in diabetics vs. non-diabetics, based on Triantafyllou et al., on the one hand, GTT was significantly longer in patients with diabetes compared with non-diabetic patients. On the other hand, SBTT was significantly shorter in diabetic compared with non-diabetic controls. Cecum was reached in 69 and 89.6% of the diabetic and non-diabetic patients, respectively (18). The difference between these two studies can be explained by the exclusion of patients with symptoms of a motility disorders in our study. Moreover, a large proportion of patients (13 out of 29) were excluded from the SBTT analysis in the above-mentioned study (18) and may have affected the findings of a faster SBTT in diabetic patients. Another important factor is the glucose level and the degree of diabetic control (HBA1c) in these patients. This data was not available on the day which patients had their VCE study. Alternatively, our study might lack sufficient statistical power to differentiate diabetic and non-diabetic patients.

Our study has some limitations. We used historical healthy controls for WMC transit measurement and obscure GI bleeding/IDA patients to approximate healthy controls for VCE measurement. Therefore, prospective-controlled studies using both WMC and VCE in a multicenter setting would be a more optimal design to draw more definite conclusions regarding the segmental transit times.

Based on our data and the body of literature, VCE could potentially be used for the measurement of SBTT in the fasting state. Although our studied population did not have any known or suspected motility disorder, validation of the method in healthy controls should be pursued.

## Author Contributions

MZ designed and supervised the study. RH, MS, and MZ collected the video capsule endoscopy data. RH, MB, MS, ZM, and MZ analyzed and interpreted the findings. RH, MB, and MZ drafted the article. IS and RM provided data related to wireless motility capsule. All authors reviewed and approved the final version of the article.

## Conflict of Interest Statement

The authors declare that the research was conducted in the absence of any commercial or financial relationships that could be construed as a potential conflict of interest.

## References

[1] Grybäck P Jacobsson H Blomquist L Schnell PO Hellström PM. Scintigraphy of the small intestine: a simplified standard for study of transit with reference to normal values. Eur J Nucl Med Mol Imaging (2002) 29:39–45. 10.1007/s00259-001-0687-z 11807605

[2] Kim SK Small intestine transit time in the normal small bowel study. Am J Roentgenol Radium Ther Nucl Med (1968) 104:522–4. 10.2214/ajr.104.3.522 5687899

[3] Hung GU Tsai CC Lin WY. Development of a new method for small bowel transit study. Ann Nucl Med (2006) 20:387–92. 10.1007/BF03027373 16922466

[4] Rao SS Camilleri M Hasler WL Maurer AH Parkman HP Saad R Evaluation of gastrointestinal transit in clinical practice: position paper of the American and European Neurogastroenterology and Motility Societies. Neurogastroenterol Motil (2011) 23:8–23. 10.1111/j.1365-2982.2010.01612.x 21138500

[5] Wang YT Mohammed SD Farmer AD Wang D Zarate N Hobson AR Regional gastrointestinal transit and pH studied in 215 healthy volunteers using the wireless motility capsule: influence of age, gender, study country and testing protocol. Aliment Pharmacol Ther (2015) 42:761–72. 10.1111/apt.13329 26223837

[6] Mustafa BF Samaan M Langmead L Khasraw M. Small bowel video capsule endoscopy: an overview. Expert Rev Gastroenterol Hepatol (2013) 7:323–9. 10.1586/egh.13.20 23639090

[7] Bouchard S Ibrahim M Van Gossum A. Video capsule endoscopy: perspectives of a revolutionary technique. World J Gastroenterol (2014) 20:17330–44. 10.3748/wjg.v20.i46.17330 25516644 PMC4265591

[8] Van de Bruaene C De Looze D Hindryckx P. Small bowel capsule endoscopy: where are we after almost 15 years of use? World J Gastrointest Endosc (2015) 7:13–36. 10.4253/wjge.v7.i1.13 25610531 PMC4295178

[9] ASGE Technology Committee Wang A Banerjee S Barth BA Bhat YM Chauhan S Wireless capsule endoscopy. Gastrointest Endosc (2013) 78:805–15. 10.1016/j.gie.2013.06.026 24119509

[10] Gerson LB Fidler JL Cave DR Leighton JA. ACG clinical guideline: diagnosis and management of small bowel bleeding. Am J Gastroenterol (2015) 110:1265–87. 10.1038/ajg.2015.246 26303132

[11] Fleischer D. Capsule imaging. Clin Gastroenterol Hepatol (2005) 3:S30–2. 10.1016/S1542-3565(05)00261-2 16012992

[12] Matsuura M Inamori M Endo H Matsuura T Kanoshima K Inoh Y Lubiprostone decreases the small bowel transit time by capsule endoscopy: an exploratory, randomised, double-blind, placebo-controlled 3-way crossover study. Gastroenterol Res Pract (2014) 2014:879595. 10.1155/2014/879595 25614738 PMC4295152

[13] Sarosiek I Selover KH Katz LA Semler JR Wilding GE Lackner JM The assessment of regional gut transit times in healthy controls and patients with gastroparesis using wireless motility technology. Aliment Pharmacol Ther (2010) 31:313–22. 10.1111/j.1365-2036.2009.04162.x 19814743 PMC4444219

[14] Takahashi T. Interdigestive migrating motor complex – its mechanism and clinical importance. J Smooth Muscle Res (2013) 49:99–111. 10.1540/jsmr.49.99 24662475 PMC5137267

[15] Velayos Jimenez B Fernandez Salazar L Aller de la Fuente R de la Calle Valverde F Del Olmo Martínez L Arranz Santos T [Study of gastrointestinal transit times with capsule endoscopy]. Gastroenterol Hepatol (2005) 28:315–20. 10.1157/13076347 15989811

[16] Kalantzis C Triantafyllou K Papadopoulos AA Alexandrakis G Rokkas T Kalantzis N Effect of three bowel preparations on video-capsule endoscopy gastric and small-bowel transit time and completeness of the examination. Scand J Gastroenterol (2007) 42:1120–6. 10.1080/00365520701251601 17710680

[17] Westerhof J Koornstra JJ Hoedemaker RA Sluiter WJ Kleibeuker JH Weersma RK. Diagnostic yield of small bowel capsule endoscopy depends on the small bowel transit time. World J Gastroenterol (2012) 18:1502–7. 10.3748/wjg.v18.i13.1502 22509082 PMC3319946

[18] Triantafyllou K Kalantzis C Papadopoulos AA Apostolopoulos P Rokkas T Kalantzis N Video-capsule endoscopy gastric and small bowel transit time and completeness of the examination in patients with diabetes mellitus. Dig Liver Dis (2007) 39:575–80. 10.1016/j.dld.2007.01.024 17433797

